# miRNA-135a promotes hepatocellular carcinoma cell migration and invasion by targeting forkhead box O1

**DOI:** 10.1186/s12935-016-0328-z

**Published:** 2016-08-02

**Authors:** Yue-Bin Zeng, Xing-Hua Liang, Guang-Xian Zhang, Nan Jiang, Tong Zhang, Jian-Ying Huang, Lei Zhang, Xian-Cheng Zeng

**Affiliations:** 1 Department of Infectious Diseases, Zengcheng People’s Hospital (BoJi-Affiliated Hospital of Sun Yat-Sen University), Zengcheng, 511300 China; 2 Department of Gastroenterology, Zengcheng People’s Hospital (BoJi-Affiliated Hospital of Sun Yat-Sen University), Zengcheng, 511300 China; 3 School of Basic Medical Sciences, Guangzhou University of Chinese Medicine, Guangzhou, 510006 China; 4 Department of Hepatic Surgery, The Third Affiliated Hospital of Sun Yat-sen University, Guangzhou, 510630 China; 5 Department of Clinical Laboratory, Zengcheng People’s Hospital, (BoJi-Affiliated Hospital of Sun Yat-Sen University), Zengcheng, 511300 China; 6 Department of General Surgery and Clinical Laboratory, Zengcheng People’s Hospital (BoJi-Affiliated Hospital of Sun Yat-Sen University), Zengcheng, 511300 China

**Keywords:** Hepatocellular carcinoma, miR-135a, FOXO1, Migration and invasion

## Abstract

**Aims:**

Hepatocellular carcinoma (HCC) is the third leading cause of cancer mortality worldwide. Many microRNAs (miRNAs), small non-coding RNAs, are involved in regulating cancer cell proliferation, metastasis, migration, invasion and apoptosis.

**Main methods:**

We investigated the expression of miR-135a in HCC cell lines and clinical tissues. The effect of miR-135a on migration and invasion of HepG2 and MHCC-97L were examined using wound healing and Transwell assay. We determined the expression of miR-135a, forkhead box O1 (FOXO1), matrix metalloproteinase-2 (MMP-2) and Snail using real-time PCR and western blotting.

**Key findings:**

We found miR-135a was upregulated in HCC cell lines and tissues. miR-135a overexpression promoted HCC cells migration and invasion, whereas miR-135a inhibition suppressed HCC cells migration and invasion. miR-135a overexpression could upregulate the expression of MMP2, Snail and the phosphorylation of AKT, but decreased FOXO3a phosporylation. Tumor suppressor FOXO1 was the direct target for miR-135a.

**Significance:**

Our results suggested that miR-135a might play an important role in promoting migration and invasion in HCC and presents a novel mechanism of miRNA-mediated direct suppression of FOXO1 in HCC cells.

**Electronic supplementary material:**

The online version of this article (doi:10.1186/s12935-016-0328-z) contains supplementary material, which is available to authorized users.

## Background

Hepatocellular carcinoma (HCC) is the fifth most common cancer and the third leading cause of cancer-related mortality worldwide [1, 2]. As most HCC patients in China are infected with hepatitis B virus (HBV) and hepatitis C virus (HCV), the incidence and mortality are higher. Chinese cases account for 55 % of all HCC cases globally [1]. Currently, the most effective therapy is surgery. Nevertheless, about 85 % of patients have locally advanced tumor or distant metastasis at diagnosis, and are not suitable candidates for surgery. Consequently, they cannot be cured by surgical resection and liver transplantation, which renders curing HCC difficult [3]. Therefore, there is an urgent need for in-depth understanding of the molecular mechanisms of HCC metastasis to identify new treatment targets.

microRNAs (miRNAs), small non-coding RNA containing 17–27 nucleotides, regulate gene expression by mediating target mRNA degradation or translational repression. Recently, it was revealed that many miRNAs play crucial roles in tumorigenesis and cancer progression [4, 5]. Most miRNAs are involved in regulating cancer cell proliferation, migration, invasion, metastasis and apoptosis. miR-135a plays important role in many tumors. In ovarian cancer, miR-135a acts as a tumor suppressor by downregulating homeobox A10 (HOXA10) expression with concomitant enhancement of caspase-3 and p53, and reduction of Bcl-2 [6, 7]. In breast cancer, miR-135a promotes tumor migration and invasion by targeting HOXA10 [8]. In classic Hodgkin lymphoma, miR-135a mediates Janus kinase 2 (JAK2) downregulation and decreases both the mRNA and protein levels of the anti-apoptotic gene Bcl-xL (BCL2L1), leading to apoptosis [9]. In HCC, miR-135a transcribed by forkhead box (FOX) M1 induces the development of portal vein tumor thrombus by promoting metastasis, and inhibiting metastasis suppressor 1 (MTSS1) [10]. However, what is/are the target gene(s) of miR-135a and how miR-135a causes metastasis in HCC remain poorly understand.

FOXO1 which is a member of the forkhead transcriptional factor family, functions as a tumor suppressor in various carcinomas. For example, breast, prostate, cervical, gastric and endometrial cancer, its downregulation accelerates tumor progression in these tumors [11–15]. FOXO1 expression also is downregulated in HCC [16]. Xu et al. [17] find that FOXO1 and FOXO3a expression are upregulated when oncomiRNA miR-96 is inhibited in HCC cells, inhibition of FOXO1 and FOXO3a promoted HCC cell proliferation and colony formation ability. However, the function of miR-135a on HCC metastasis has not been reported.

In the current study, we determined miR-135a expression in HCC cells, normal liver cells, HCC tissues and adjacent normal live tissues and investigated the effect of miR-135a overexpression on HCC cell migration and invasion. miR-135a likely induced HCC cell metastasis and invasion by directly targeting the 3′ untranslated region (3′ UTR) of FOXO1 mRNA, consequently increasing Snail and matrix metalloproteinase-2 (MMP2) expression, inhibiting FOXO3a phosphorylation, and promoting AKT phosphorylation. Our results suggest that miR-135a may play an important role in the development and progression of HCC.

## Methods

### Cell culture and transfection

Human HCC cell lines Bel-7402, Huh7, HepG2, MHCC-97H, MHCC-97L and SMMC-7721 and the immortalized normal liver epithelial cell LO2 were obtained from ATTC. HCC cells was maintained in Dulbecco’s modified Eagle’s medium (Invitrogen, Carlsbad, CA, USA) supplemented with 10 % fetal bovine serum (FBS; HyClone, Logan, UT, USA). LO2 cells were cultured in bronchial epithelial growth medium (Clonetics Corporation, Walkersville, MD, USA), supplemented with 5 ng/ml epithelial growth factor, 70 ng/ml phosphoethanolamine, and 10 % FBS. Cells were maintained in a humidified atmosphere at 37 °C with 5 % CO_2_.

miR-135a mimic, miR-135a inhibitor, and their negative control were purchased from RiboBio Co (Guangzhou, Guangdong, China). 20 nm olignonucleotides were transfected into cells using Lipofectamine RNAiMAX according to the manufacturer’s instruction.

### Wound healing assay

One day before the wound healing assay was performed, stable HepG2 and MHCC-97L cells were trypsinized and seeded equally in 6-well cell culture plates, and grew to almost total confluence in 24 h. We created an artificial homogenous wound in the monolayer using a sterile 100-μl pipette tip. After scratching, the cells were washed with serum-free medium. Images of cells migrating into the wound were captured at 0 and 24 h using an inverted microscope (40×).

### In vitro invasion assay

We performed the invasion assay using Transwell chambers containing Matrigel-coated 8-μm membrane filter inserts. Cells were trypsinized and suspended in serum-free medium. Then, 1.5 × 10^5^ cells were added to the top chamber, whereas the bottom chamber was filled with medium containing 10 % FBS. After 48-h incubation, cells that had invaded through the membrane to the lower surface were fixed with 4 % paraformaldehyde, stained with hematoxylin, and counted under ×100 magnification.

### RNA extraction and real-time quantitative PCR

Total RNA from human HCC cell lines (Bel-7402, Huh7, HepG2, MHCC-97H, MHCC-97L, SMMC-7721) and primary tumor tissues was extracted using TRIzol according to the manufacturer’s instructions (Invitrogen). The extracted RNA was dissolved with RNase-free water pretreated with diethyl phosphorocyanidate. Complementary DNA was synthesized from total RNA using reverse transcription–PCR (RT-PCR) with specific primers. Expression data were normalized to the geometric mean of the housekeeping gene glyceraldehyde-3-phosphate dehydrogenase (*GAPDH*) to control the variability of expression. miRNA expression was defined based on the threshold cycle (Ct), and relative expression levels were calculated as 2^−[(Ct of miR−135a) − Ct of U6]^ after normalization with reference to U6 small nuclear RNA expression. The primers for RT-PCR and real-time PCR were designed using Primer Express Software V2.0 (Applied Biosystems, Foster City, CA, USA).

### Western blotting

Equal amounts of protein were separated by electrophoresis on a 10 % sodium dodecyl sulfate–polyacrylamide gel and electrotransferred to a nitrocellulose membrane. After blocking with 5 % non-fat milk solution in Tris-buffered saline with Tween20 (TBST) for 1 h, the membrane was incubated with primary antibodies for FOXO3a, phosphorylated (p)-FOXO3a, AKT, p-AKT, MMP2, and Snail for 1.5–2 h at room temperature. Anti–β-actin antibody was used as the internal loading control.

### Luciferase assay

Cells (5 × 10^5^) were seeded in triplicate in 6-well plates and allowed to settle for 24 h. Luciferase plasmid pGL3-FOXO-3′UTR (wild-type/mutant, wt/mu) or control luciferase plasmid plus 1 ng pRL-TK *Renilla* plasmid (Promega, Madison, WI, USA) were transfected into HepG2 cells using Lipofectamine 2000 according to the manufacturer’s recommendation (Invitrogen). Luciferase and *Renilla* signals were measured 48 h after transfection using a Dual Luciferase Reporter Assay Kit according to the manufacturer’s protocol (Promega). Three independent experiments were performed and the data are presented as the mean ± SD.

### Statistical analysis

Statistical analysis was performed using SPSS 13.0 statistical software package, Student *t* test was used to evaluate significant differences between two groups of data in all pertinent experiments. p < 0.05 (2-tailed paired *t*-test) was considered significantly different for two groups of data.

## Results

### miR-135a was upregulated in HCC cell lines and tissues

**Fig. 1:**
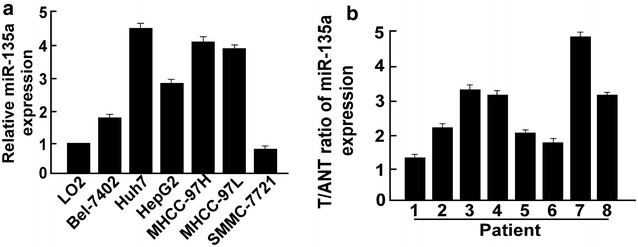
miR-135a is upregulated in HCC cells and tissues. **a** Real-time PCR analysis of miR-135a expression in normal liver cells (LO2) and HCC cell lines Bel-7402, Huh7, HepG2, MHCC-97H, MHCC-97L, and SMMC-7721. **b** miR-135a expression in eight paired HCC tissues (T) and their adjacent normal tissues (ANT). Average miR-135a expression was normalized to U6 expression. Bars represent the mean of three independent experiments. *p < 0.05

Real-time quantitative PCR assay revealed that miR-135a was significantly upregulated in the six HCC cell lines (Bel-7402, Huh7, HepG2, MHCC-97H, MHCC-97L and SMMC-7721) compared with that of in normal LO2 liver cells (Fig. 1a). We further analyzed miR-135a expression in HCC tissues (T) and adjacent normal liver tissues (ANT), suggesting miR-135a was upregualted in HCC tissues compared to adjacent normal live tissues (Fig. 1b). These suggested that miR-135a was upregulated in hepatocellular carcinoma, it might be an oncomiRNA.

### miR-135a regulates HCC cell migration and invasion

**Fig. 2:**
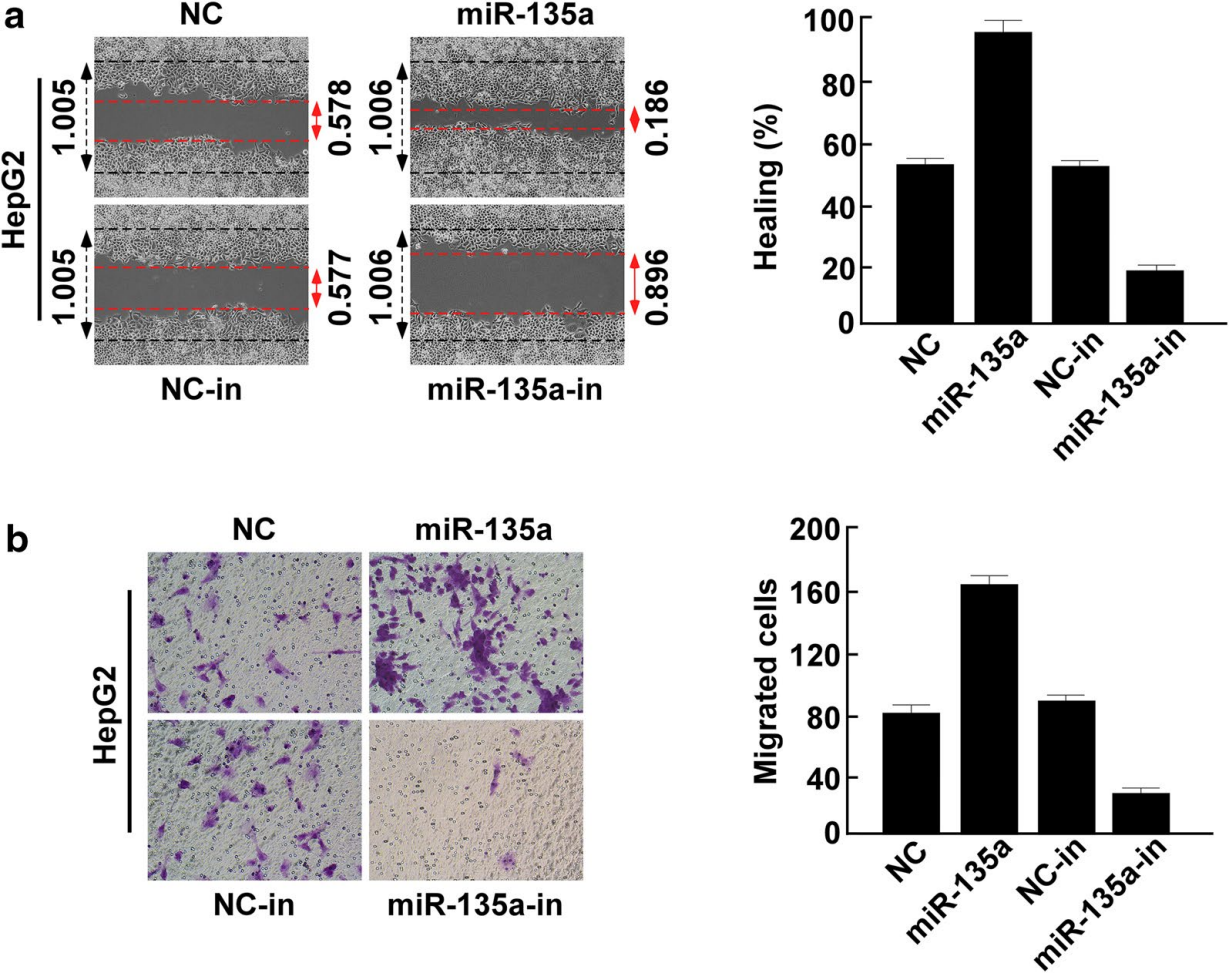
Ectopic expression of miR-135a promoted HCC cell metastasis and increased adhesion. **a** Wound healing determined the effect of miR-135a on cell migration by modulating miR-135a expression. **b** Transwell invasion assay determined the effect of miR-135a on cell invasion by modulating miR-135a expression. *Error bars* represent mean ± SD of three independent experiment

To investigate the biological role of miR-135a in the development and progression of HCC, we transfected miR-135a mimic or control into HepG2 and MHCC-97L to determine its effect on cellular migration and invasion. miR-135a overexpression dramatically increased the HCC cell migration compared with that of in negative control (NC)-transfected cells. When miR-135a was inhibited, the HCC cell migration was decreased compared to that of in NC (NC-in) group (Fig. 2a and Additional file 1: Figure S1A). The Transwell invasion assay revealed that miR-135a overexpression promoted HCC cell invasion, Inhibition of miR-135a suppressed HCC cell invasion (Fig. 2b and Additional file 1: Figure S1B). Therefore, overexpression of miR-135a promoted HCC cell migration and invasion, inhibition of miR-135a suppressed HCC cell migration and invasion.

### miR-135a regulates metastasis-associated genes expression

**Fig. 3:**
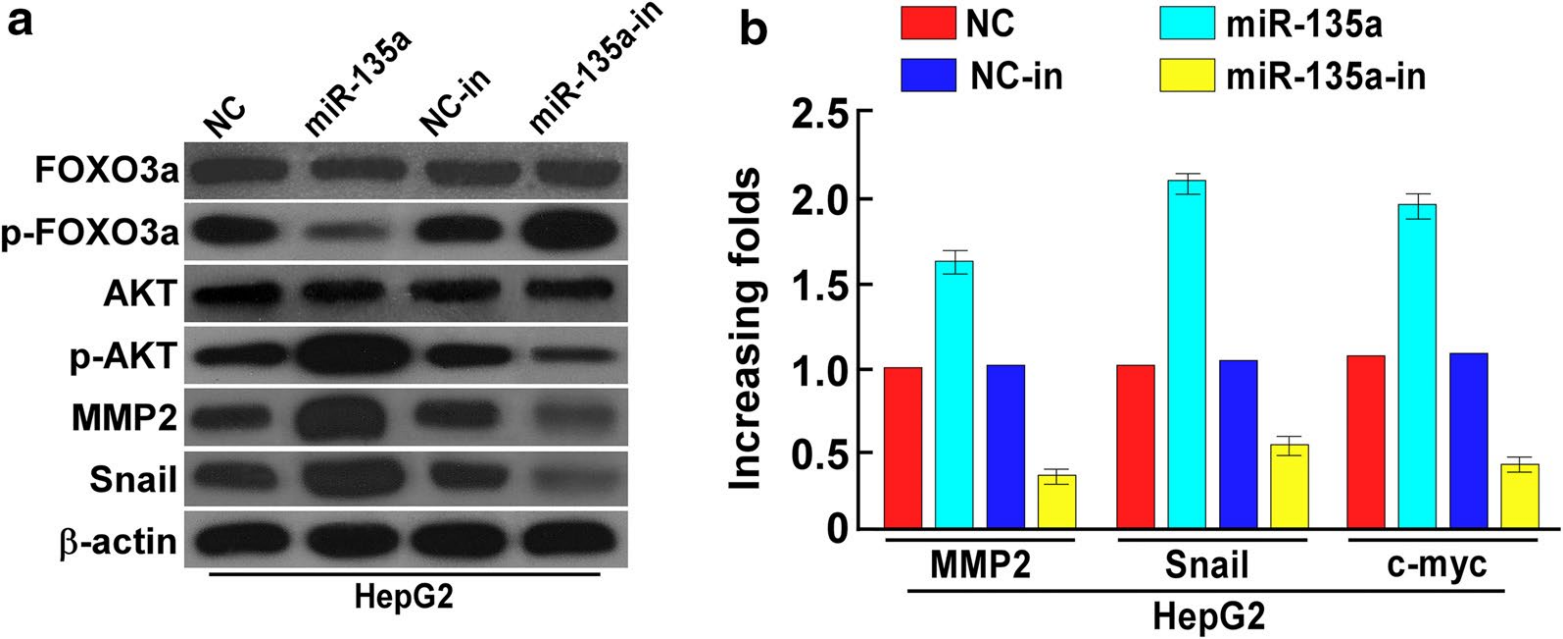
miR-135a induced migration and invasion by increasing the expression metastasis-associated genes. **a** Western blots of FOXO3a, p-FOXO3a, AKT, p-AKT, MMP2 and Snail in HepG2 cells transfected with miR-135a mimic or miR-135a inhibitor. β-actin was used as the loading control. **b** Real-time PCR analysis of MMP2 and Snail expression in HepG2 cells. *Error bars* represent mean ± SD of three independent experiment. *p < 0.05

Activation PI3 K/AKT pathway plays critical role in tumor metastasis [18], FOXO3a activation leads to transactivation of the E-cadherin expression and inhibit EMT associated genes expression. Phosphorylation of FOXO3a induces its nuclear export, and promotes FOXO3a degradation [19]. Snail and MMP2 promote tumor invasion and metastasis. Western blotting analysis revealed that miR-135a upregulation inhibited FOXO3a phosphorylation and increased AKT phosphorylation and MMP2 and Snail expression, knockdown of miR-135a promoted the phosphorylation of FOXO3a and inhibited AKT phosphorylation and the expression of MMP2 and Snail (Fig. 3a and Additional file 2: Figure S2A). Luciferase analysis revealed that miR-135a upregulation promoted the transcription of the metastasis-associated genes MMP2 and Snail, vice verse (Fig. 3b and Additional file 2: Figure S2B).

### miR-135a directly targeted FOXO1 in HCC cells

**Fig. 4:**
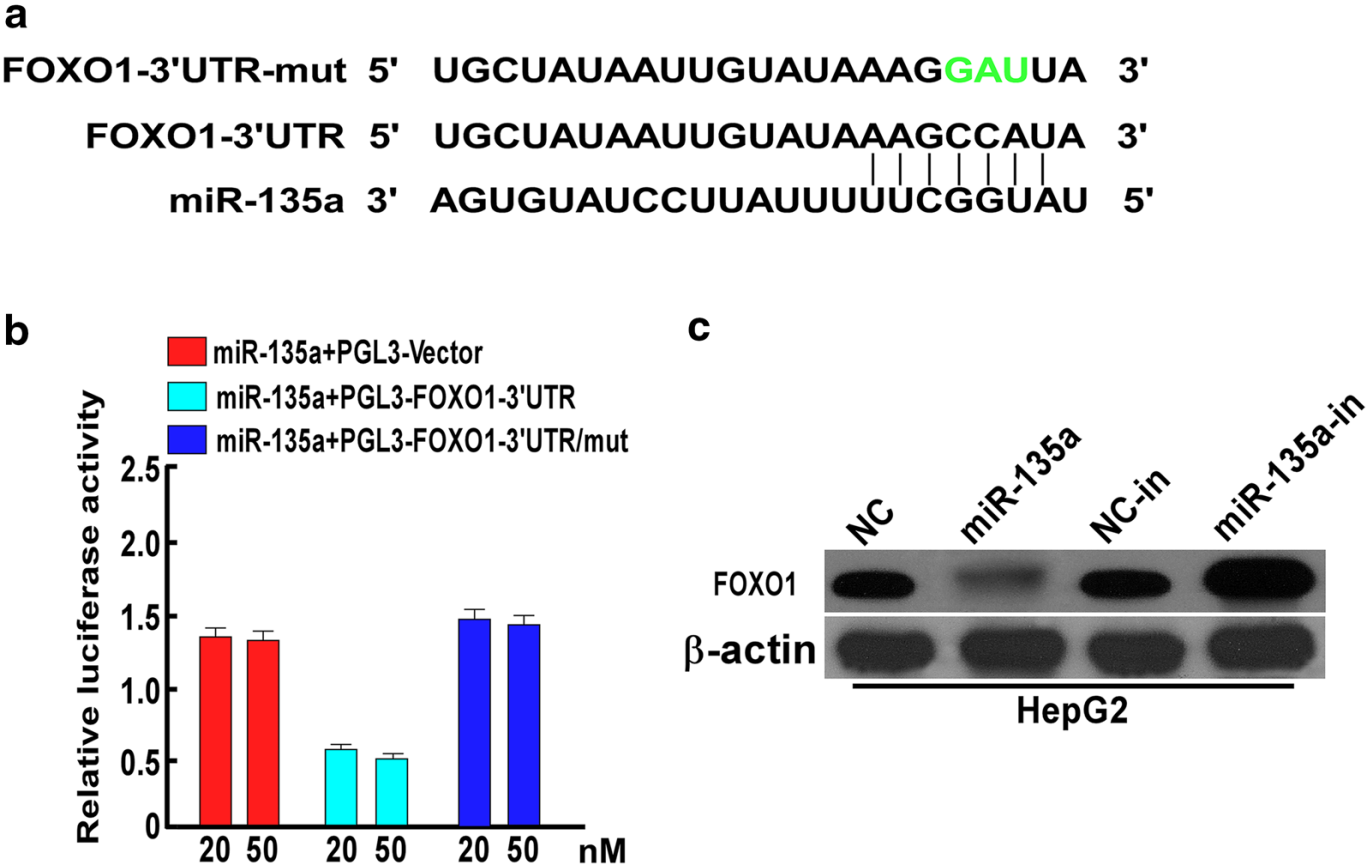
miR-135a downregulated FOXO1 by directly targeting the FOXO1 3′ UTR. **a** Predicted miR-135a target sequences in 3′ UTR of FOXO1 (FOXO1-3′UTR) and mutant containing three mutated nucleotides (in *green*) in FOXO1-3′UTR (FOXO1-3′UTR-mut). **b** Luciferase assays of HepG2 cells transfected with pGL3 control, pGL3-FOXO1-3′UTR, or pGL3-FOXO1-3′UTR-mut reporter and 20 nM or 50 nM miR-135a mimic. **c** Western blot of FOXO1 expression after miR-135a overexpression or knockdown on HepG2. *Error bars* represent mean ± SD of three independent experiments. *p < 0.05

We used TargetScan, PicTar and miRBase to predict the target genes of miR-135a, and found that FOXO1 was one of the candidates, the potential binding sequence for miR-135a was identified in the 3′UTR of FOXO1 (Fig. 4a). We also observed a consistent and dose-dependent reduction of luciferase activity upon miR-135a transfection in HepG2 cells (Fig. 4b). As predicted, ectopic expression of miR-135a in the HepG2 cells decreased FOXO1 expression, while miR-135a-in transfection increased its expression (Fig. 4c). We also determined FOXO1 expression in normal LO2 liver cells and HCC cells, real-time quantitative PCR assay found the mRNA level of FOXO1 in LO2 was the same as HCC cells (Additional file 3: Figure S3), but FOXO1 was downregulated in protein level in HCC cells (Additional file 4: Figure S4C), suggesting miR-135a inhibited the translation of FOXO1. We also determined the role of miR-35a in LO2 cells, and found knockdown of LO2 slightly inhibited the migration and invasion of cells (Additional file 4: Figure S4A, B).

## Discussion

In the present study, miR-135a expression was upregulated in HCC cells and HCC tissue. Our findings indicate that ectopic expression of miR-135a enhanced HCC cell migration and invasion, while miR-135a inhibition reduced these effects. Moreover, we proved that miR-135a upregulation in HCC cells led to overexpression of the metastasis-associated genes MMP2 and SNAI1, and the phosphorylation of AKT, and increased FOXO3a phosphorylation. The luciferase assay indicated that miR-135a downregulates FOXO1 by targeting its 3′ UTR directly. These findings suggested that miR-135a upregulation might play an important role in promoting carcinogenesis and progression of HCC.

FOXO1 is a famous tumor suppressor gene, and regulates cell proliferation, invasion, metastasis and apoptosis. For example, Lee et al. [20] reported that inhibition of Aurora A by RNA interference in HCC cells upregulated FOXO1 in a p53-dependent manner, which induces cell cycle arrest. Wang et al. [21] demonstrated that miR-145 suppresses HCC by downregulating insulin receptor substrate-1 (IRS1) and inhibiting the downstream AKT/FOXO1 signaling.

FOXO1 also plays a role in metastasis and invasion in T lymphocyte cancer, rhabdomyosarcoma, ovarian cancer, breast cancer, and prostate cancer. FOXO1 targeting of family with sequence similarity 65 member B (Fam65b) tonically dampens chemokine-induced migration by repressing Ras homolog family member A (RhoA) activity in T lymphocytes [22]. In breast cancer, cell division cycle 25A (CDC25A) mediates metastasis by regulating MMP1 through FOXO1 [23]. In prostate cancer, FOXO1 inactivation due to frequent loss of phosphatase and tensin homolog (PTEN) in prostate cancer cells may render the oncogenic activities of runt-related transcription factor 2 (Runx2) unchecked, thereby driving promiscuous expression of Runx2 target genes involved in cell migration and invasion and favoring prostate cancer progression [24]. Recently, Wilson K.C. Leung and colleagues found Wnt/β-Catenin can activate miR-183/96/182 to inhibit FOXO1 to promote the invasion and metastasis of HCC [25], this suggesting FOXO1 inhibits HCC invasion and metastasis.

In this study, we demonstrated that FOXO1 is also an miR-135a target that promotes HCC metastasis and presents a novel mechanism of miRNA-mediated FOXO1 downregulation. Bioinformatics analysis indicated that the tumor suppressor *FOXO1* is a theoretical target gene of miR-135a. The wound healing and Transwell assays showed that miR-135a overexpression promoted HCC cell migration and invasion. Western blotting and real-time PCR revealed that metastasis-related genes were upregulated in HCC cells transfected with miR-135a mimic, while miR-135a inhibition decreased the expression of the metastasis-related genes. The luciferase activity assay and point mutation analysis demonstrated that miR-135a mediated FOXO1 downregulation by binding to the FOXO1 3′ UTR. This suggested that the biological function of miR-135a was against FOXO1 to inhibit HCC cell migration and invasion. Nevertheless, one limitation in this study was that we were unable to clarify the particular mechanism of miR-135a facilitation of HCC metastasis and the related signaling pathway. Therefore, further research was required to explore the biological function of miR-135a.

## Conclusion

miR-135a is an oncogenic miRNA in HCC and promotes HCC cell migration and invasion, which plays a role in HCC progression. Complete understanding of its biological function will aid in the discovery of a novel therapeutic target for treating HCC.

## Additional files

10.1186/s12935-016-0328-z Ectopic expression of miR-135a promoted HCC cell metastasis and increased adhesion in MHCC-97L. A. Wound healing determined the effect of miR-135a on cell migration by modulating miR-135a expression. B. Transwell invasion assay determined the effect of miR-135a on cell invasion by modulating miR-135a expression. Error bars represent mean ± SD of three independent experiment.
10.1186/s12935-016-0328-z miR-135a induced migration and invasion by increasing the expression metastasis-associated genes. A. Western blot analysis of FOXO3a, p-FOXO3a, AKT, p-AKT, MMP2 and Snail in MHCC-97L cells transfected with miR-135a mimic or miR-135a inhibitor. β-actin was used as the loading control. B. Real-time PCR analysis of MMP2 and Snail expression in MHCC-97L cells. Error bars represent mean ± SD of three independent experiment. *p < 0.05.
10.1186/s12935-016-0328-z Real-time PCR determined FOXO1 expression in normal live cells and HCC cells.
10.1186/s12935-016-0328-z The effect of miR-135a on migration and invasion of LO2. A. A. Wound healing determined the effect of miR-135a on migration of LO2 by inhibiting miR-135a expression. B. Transwell invasion assay determined the effect of miR-135a on invasion of LO2 by inhibiting miR-135a expression. C. Western blot determined FOXO1 expression in LO2 cells, HepG2 cells and HepG2 cells transfected miR-135a inhibitor. β-actin was used as the loading control. Error bars represent mean ± SD of three independent experiment.
